# Drinking water access and quality in the Gaza Strip prior to 7 October 2023 and implications for reconstruction

**DOI:** 10.1186/s12940-025-01191-6

**Published:** 2025-07-01

**Authors:** Curdin Brugger, Branwen Nia Owen, Bassam Abu Hamad, Tammo van Gastel, Federico Sittaro, Rodolfo Rossi, Nicole Probst-Hensch, Mirko S. Winkler

**Affiliations:** 1 Department of Epidemiology and Public Health, Swiss Tropical and Public Health Institute, Kreuzstrasse 2, Allschwil, 4123 Switzerland; 2 University of Basel, Petersplatz 1, Basel, 4001 Switzerland; 3 Al Quds University, Gaza City, Palestine; 4 International Committee of the Red Cross, 19 Avenue de la Paix, Geneva, 1202 Switzerland; 5 Department of Environmental Systems Science, ETH Zürich, Universitätstrasse 16, Zürich, 8092 Switzerland

**Keywords:** Drinking water, Household survey, Protracted conflict, Water quality, Water insecurity

## Abstract

**Background:**

The water supply of the Gaza Strip has been unstable and under great strain for decades, resulting in major problems with the quality, reliability, and acceptability of drinking water. Destruction of water infrastructure and concerns over the quality of piped water have resulted in a complex constellation of drinking water sources. We aim to describe the different types of drinking water sources used by households, compare water quality from drinking water samples, present different water treatments used in households and highlight different insecurities around water access in households.

**Methods:**

We conducted a cross-sectional household survey in North Gaza, Gaza and Rafah between January and March 2023. Using an interviewer-administered survey, we collected information on drinking water sources and insecurities and obtained a drinking water sample from the tap in the household. The water samples were analyzed for microbial contamination, nitrate, sodium and mineral content.

**Results:**

We collected data from 905 households. Only 3% had access to a single water source, 87% had access to two sources and 96% had access to piped water from the municipality. Piped municipal water was mainly used for hygiene and bathing, while the three most used sources for drinking were tanker trucks (82%), public taps (10%) and piped water from the municipalities (3.7%). Fecal coliform was present in 20% of water samples, 1% had high nitrate levels and nearly all samples had low mineral content. While around 15–19% of the households were sometimes or often water insecure, over 90% reported never drinking from undesirable sources, drinking unsafe water, or going to sleep thirsty. Households using municipal piped water tended to be most water secure.

**Conclusions:**

The water quality and insecurity about accessibility and quality of water pose a health threat and need to be addressed at system level. Rebuilding the water infrastructure will be a key element during the reconstruction after the current war. It is crucial that the shortcomings of the pre-war water system are not rebuilt, and lessons are learnt from pre-war data to establish a health-promoting water system in the Gaza Strip.

**Clinical trial number:**

Not applicable.

**Supplementary Information:**

The online version contains supplementary material available at 10.1186/s12940-025-01191-6.

## Background

**Fig. 1:**
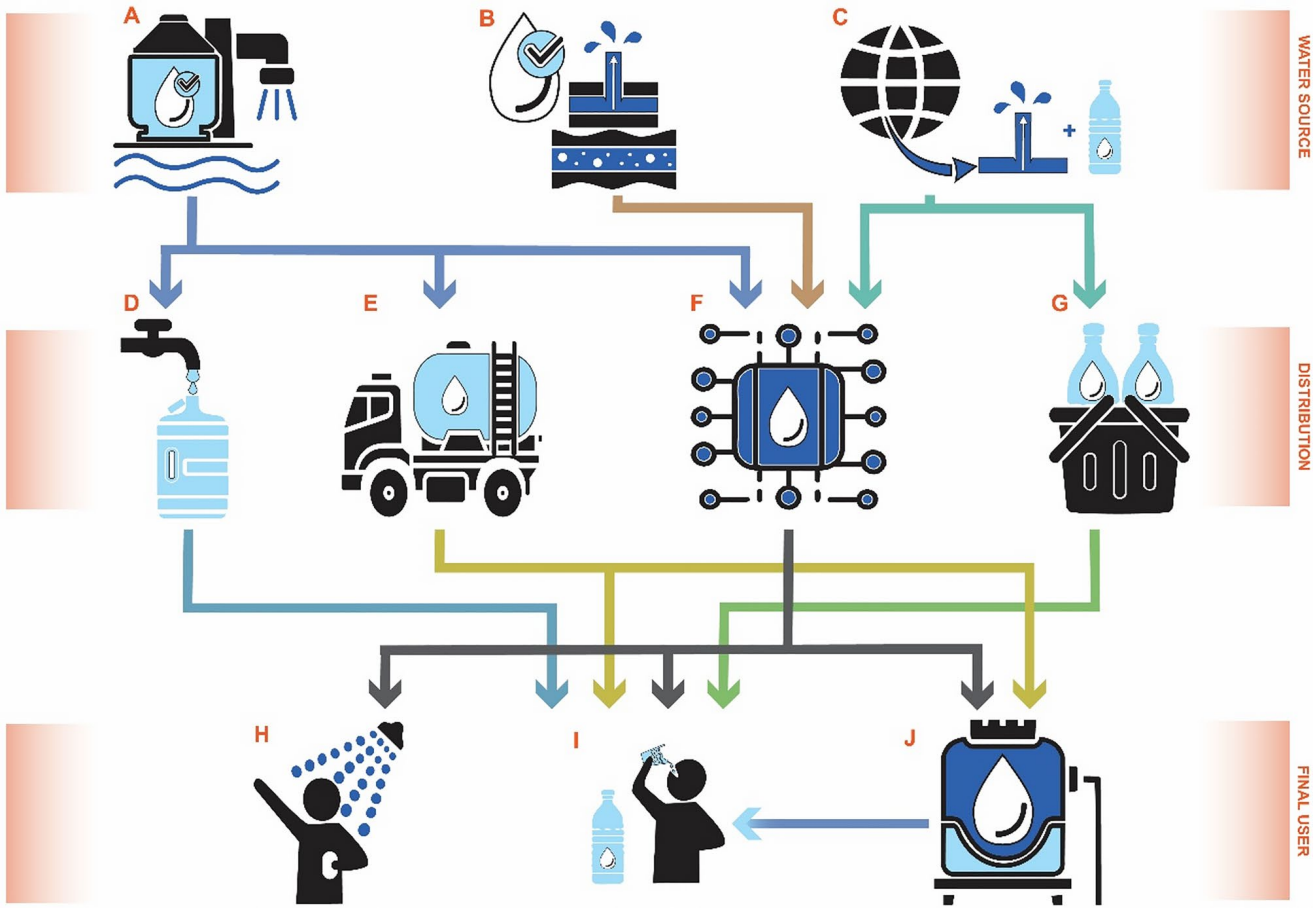
Schematic diagram of the Gaza Strip water supply system. Water sources: **A** = Desalination plants, **B** = Groundwater, and **C** = Imported water sources. Distribution: **D** = Public taps and sabeels, **E** = Tanker trucks, **F** = Public piped water network, and **G** = Shops and water vendors. Final users: **H** = Showering and bathing, **I** = Human consumption, and **J** = Storage in the household for different usages (Figure by Aline Veillat, adapted from the Palestinian Water Authority [2])

The water quality has decreased drastically in the Gaza Strip over the past two decades. While nearly all residents had access to improved water sources 25 years ago, 25–30% of Gazans did not have regular access to running water in 2018 [1]. Destruction of water infrastructure and concerns over the quality of piped water have resulted in a complex water supply system with different types of water sources and distribution networks being used. Numerous actors produce drinking water, resulting in various systems of water transport and distribution, making it a formidable challenge to have a clear overview of the drinking water system and its quality. Figure 1 shows a simplified schematic overview of the different water sources and distribution paths in the Gaza Strip. Household drinking water in the Gaza Strip primarily comes from desalination plants (Fig. 1A) and groundwater sources (Fig. 1B), or is piped from Israel (Fig. 1C) [2]. Other sources used by some households in the Gaza Strip include imported bottled water (Fig. 1C), water piped from desalination plants from private companies into houses, small-scale vendors, protected wells and rainwater [2, 3]. The main ways of delivering water from the production source to the households for consumption are tanker trucks (Fig. 1E), piped water from the municipality (Fig. 1F) and public taps (Fig. 1D) [3]. Water delivered to households by tanker trucks is mainly produced by small-scale brackish water desalination plants owned by the public and private sector, as well as some non-governmental organizations [4, 5]. Water piped to the households by the municipalities originates from different water sources such as municipal wells, desalination plants and piped water from Israel. The proportion of the different sources in the piped water can vary depending on the geographic area and the availability of water from the sources throughout the year [6, 7]. Public taps, also called Sabeel water (Fig. 1D), are frequently attached to mosques, hospitals or organizations. They are established as a charity in streets and neighborhoods. Small desalination units produce and provide water for the neighborhood free of charge.

Because of the diverse and weak infrastructure, there are major issues and concerns with drinking water quality [4]. Water quality in the Gaza Strip is primarily assessed at its source (groundwater wells or desalination plants), often neglecting transportation, storage and potential treatment in the households (e.g. water filters or boiling of water). Studies from 2014 to 2021 regarding drinking water in the Gaza Strip found high levels of microbial contamination, including fecal coliform in piped water and water from desalination plants [8, 9]. Fecal contamination poses a serious risk for the transmission of water-borne diseases [10], which is further complicated by the presence of antimicrobial resistant pathogens in the Gaza Strip [11–15]. Low mineral content in drinking water has been found to have potential negative health impacts ranging from an increased risk of cardiovascular disease [16–21], to increased risk of caries [22, 23], osteoporosis [24–26] and potentially various cancers [27–32]. This could be relevant in the Gaza Strip because of the high consumption of desalinated water [3], but data on mineral content in drinking water is lacking. Seawater intrusion and wastewater infiltrating to the aquifer are leading to high levels of nitrate, sodium and chloride in groundwater of the Gaza Strip. In 2017, only about one in nine groundwater wells was within the World Health Organization (WHO) limits in terms of nitrate and one in five wells was within the WHO limits for chloride [6]. Over the past 20 years, measured salinity levels had increased by a third in the groundwater, while there is no clear trend for nitrate [6, 33]. Excess intake of nitrate through food or drinking water has been associated with various negative health outcomes. While the evidence for infant methemoglobinemia and thyroid diseases is stronger [34–36], there are also studies showing an association of high nitrate intake and colorectal cancer and central nervous system birth defects [34]. Consumption of high levels of sodium was found to be associated with cardiovascular diseases, especially hypertension, stomach cancer, osteoporosis, renal stones and kidney disease [37–39]. Water parameters like pH, total dissolved solids, hardness and electrical conductivity, while not direct health risks, can affect water aesthetics, potentially prompting people to use alternative sources which might pose greater risks such as microbial contamination or high levels of chemicals [40–45]. Water access insecurity, concerns about quality and high costs can contribute to stress and mental health issues [46–48].

On top of the many existing challenges in terms of water quality and availability in Gaza Strip, which the ongoing war has further exacerbated, global climate change is anticipated to put additional strains on water resources in the region. Indeed, it is expected that by 2050 the number of days above 30 °C will increase by 60%, the mean temperature will increase by 1–1.5 °C and seawater intrusion will aggravate due to rising sea levels [49, 50]. Annual rainfall is estimated to reduce by up to 20%, while extreme weather such as drought or extreme rainfall and flooding will occur more frequently [49, 51].

In summary, many challenges in the water sector in the Gaza Strip can negatively impact public health, while climate change will further strain the system. At the same time, future violent escalations, such as the current war, will further impair the water system. To develop targeted interventions to prepare the water system for these challenges it is necessary to better understand water source utilization and quality in the Gaza Strip. This paper aims to describe the different drinking water sources used by households in the Gaza Strip, to present water quality measurements from household drinking water samples, compare water quality among different water sources and water insecurities of the households. Lastly, we aim to discuss lessons learnt that can inform the reconstruction of the water system in the Gaza Strip from a public health perspective after the current war and beyond.

## Methods

This study is part of a comprehensive health impact assessment (HIA) of selected essential services (water supply, wastewater management, food supply and energy supply) of the Gaza Strip. The HIA was initiated by the International Committee of the Red Cross (ICRC) in 2019 to better understand public health impacts of essential services and to prioritize and adapt their activities from a public health perspective. To address part of the evidence gaps identified in the initial stage of the HIA, we conducted a household survey, comprising of the following components: (i) questionnaire on health status and risk factors; (ii) blood pressure measurements and (iii) drinking water samples [52]. The research protocol containing detailed methodology for the complete household survey was published previously [52]. Here we present the methodology applied for the collection of the drinking water sample and the water-related section of the questionnaire. We obtained ethical clearance in the Gaza Strip, Switzerland and the ICRC’s internal ethics review board (see declarations).

### Study area and sampling

The study areas of the present survey were Gaza City, Rafah and North Gaza, which captured the diversity of settlements and water source characteristics in the Gaza Strip. Gaza City, the economic and administrative center, had a socioeconomically diverse population and a high population density, which put a significant strain on water delivery systems [2, 53]. Rafah is the southernmost governorate of the Gaza Strip and was home to many refugees and rural populations, with a large portion of residents living in poverty. North Gaza, which was frequently affected by escalations with Israel, also faced severe socio-economic challenges, including high poverty rates, making it vulnerable to health effects of poor water quality [53]. The groundwater in North Gaza had high levels of nitrate due to ongoing wastewater irrigation projects linked to the main wastewater collection facility, along with a high proportion of agricultural land [6]. Seawater intrusion into the aquifer is a major concern in Rafah and North Gaza, leading to some of the worst water quality parameters in the Gaza Strip [6, 54].

Within the three study areas, we used stratified random sampling to select households from the list of a previously conducted household survey implemented in 2020. The 2020 survey was a representative, cross-sectional household survey of people older than 40 years living in all five governorates of the Gaza Strip with a focus on non-communicable diseases [55, 56]. Revisiting the same households as a previous survey allowed us to collect data at two time points for important indicators which will be used for longitudinal analysis in separate papers. For the present paper we only use data collected during the 2023 survey.

With the sample size calculation, we aimed to be able to estimate the prevalence of various health outcomes, assuming a conservative prevalence of 50% [52]. We randomly selected 905 households, with the selection proportionate to the population size in the three study areas. The household survey started on January 24th 2023 and was completed on March 7th 2023. Before conducting the interviews and collecting the water sample, we obtained informed consent from all participants.

### Data collection

#### Drinking water sampling

After the interviews, we collected a drinking water sample from the household’s main drinking water source. The Coastal Municipalities Water Utility laboratory in Gaza City tested the samples for nine common water quality parameters (Table 1). In the results section, we present the proportion of samples that were either within or outside the limits defined by the Palestinian water quality guidelines listed in Table 1. Approximately 100 ml of water was collected in sterile containers, stored on ice in a cool box at 4–6 °C, transported to the laboratory within six hours, and analyzed within 30 h of collection. The laboratory used the analytical methods for the required parameters according to the American Public Health Association, American Water Works Association and Water Environment Federation’s *Standard methods for the examination of water and wastewater (21st edition)* [57]. A detailed description of the used methods can be found in Supplementary Table ST 1, Additional File 1. For quality control, we collected two duplicate samples from 10% of the households. The duplicate samples were collected simultaneously, adhering to the same protocol as the samples analyzed in the Coastal Municipalities Water Utility laboratory. The duplicate samples were analyzed at two independent laboratories: one sample at the Islamic University laboratory and the second at the Ministry of Health laboratory. We defined a relative percent difference of up to 20% as acceptable. The calculated relative percent difference remained below 20% for all samples tested at both laboratories, with the highest calculated value being 15%, confirming the precision of the water analysis results. As an additional quality control measure, we provided the Coastal Municipalities Water Utility laboratory with blinded duplicate and blinded blank samples. The analyzed blank samples were below the detection limits for all parameters, and the relative percent difference for all duplicate samples remained below 20%.

**Table 1:** Overview of measured water quality parameters including the Palestinian guidelines value we used to compare the results of the samples to

Water quality parameter	Abbreviation	Unit	Palestinian guidelines
Total coliform	TC	CFU/100 ml	Maximum = 3^a, b^
Fecal coliform	FC	CFU/100 ml	Maximum = 0^a, b^
Calcium	Ca^2+^	mg/L	Range = 30–200^b^
Magnesium	Mg^2+^	mg/L	Range = 10–50^b^
Nitrate	NO_3_-N	mg/L	Maximum = 50^a, b^
Sodium	Na^+^	mg/L	Maximum = 200^a, b^
pH	pH	pH units	Range = 6.5–9.5^a^
Total dissolved solids	TDS	mg/L	Range = 100–1000^a, b^
Electrical conductivity	EC	µS/cm	Recommended = 2’500^a^

^a^: Mandatory Technical Instructions: Water prepared for human consumption, PWA 2023 [58]

^b^ Palestinian Drinking Water Standards, 2010 [59]

**Fig. 2:**
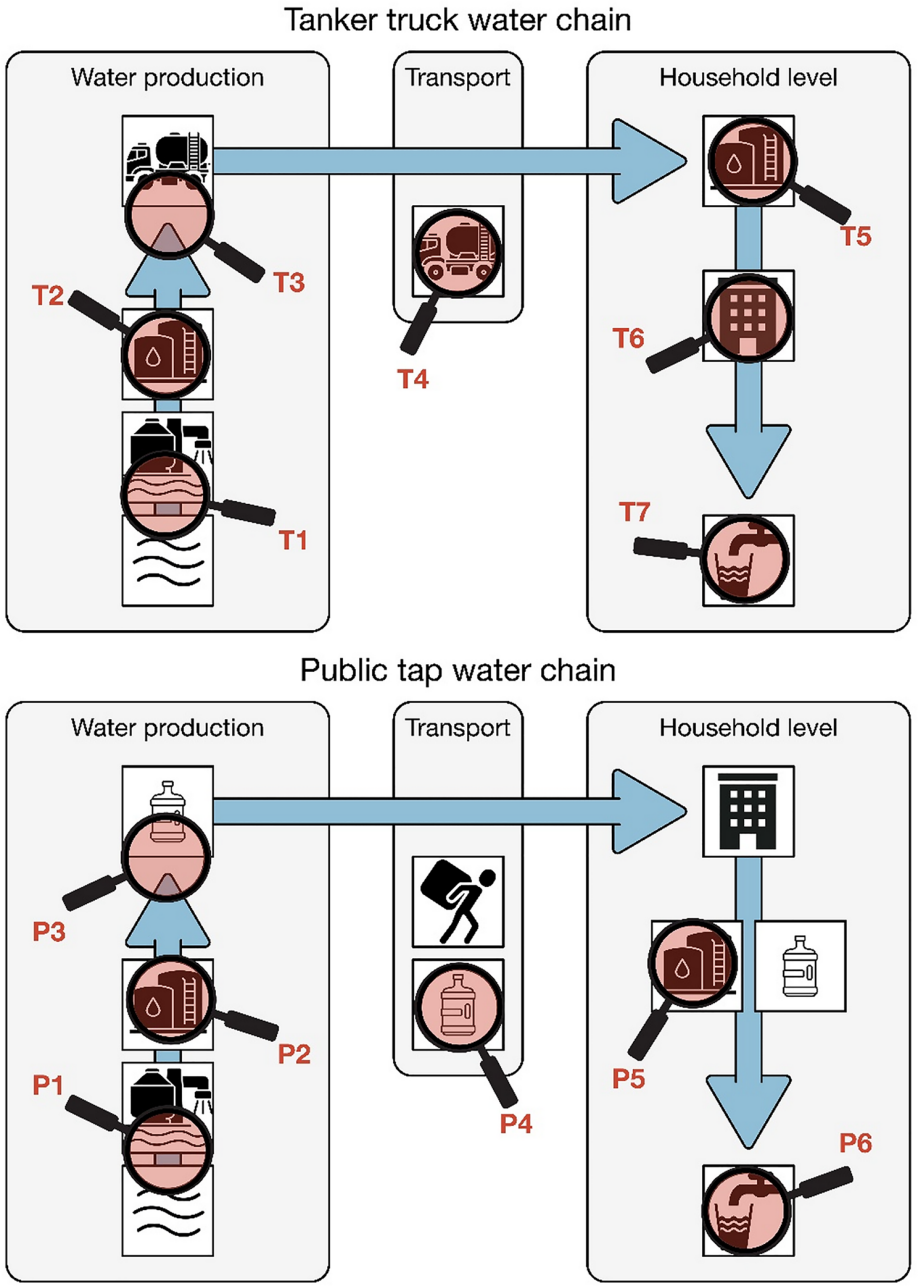
Schematic overview of the water chains and sampling locations. Sampling locations are indicated by the magnifying classes. Sampling locations in the tanker truck water chain were: T1 = Inside desalination plant before desalination, T2 = Tap or water storage tank inside desalination plant, T3 = Pipe used to fill tanker trucks, T4 = tanker trucks (Large pipe, small pipe and/or tank itself), T5 = Storage tank at the household, T6 = Tank tap inside the household and T7 = Cup or bottle used for drinking. Sampling locations in the public tap water chain were: P1 = Inside desalination plant before desalination, P2 = Tap or water storage tank inside the building / mosque, P3 = Public tap or Sabeel, P4 = Container used to transport water to the household, P5 = Container used for storage at the household if different from transportation container and P6 = Cup or bottle used for drinking

Based on the initial results, 90 households with fecal coliforms were selected for resampling. The resampling selection was based on high fecal coliform counts, geographic distribution and drinking water source. We collected a second sample three to four weeks after the first sample and analyzed it for bacteria and antimicrobial resistance at the laboratory of the Islamic University of Gaza in Gaza City. Results on antimicrobial resistance found in the second water sample haven been reported elsewhere. To better understand the diverse drinking water delivery systems and to identify different contamination locations, we selected 10 of the 90 households for additional sampling along the water distribution chain. We collected water samples at the household’s primary water source and additional samples at each potential contamination site along the water chain. In this sub-sample, we included households using water from tanker trucks (*n* = 7) and water from public taps (*n* = 3). The number and location of water samples taken varied depending on the drinking water source (Fig. 2).

#### Interviews

As part of the household survey, we interviewed residents on health status (e.g. mental health and non-communicable diseases), health determinants (e.g. water sources, recreational activities and smoking), consumption of antibiotics and knowledge about antimicrobial resistance as well as attitudes toward wastewater irrigated crops and perception of the environment. A large part of the survey focused on water-related topics such as water sources, storage and consumption, water availability and access, and wastewater reuse and antibiotic use. We built the survey on different pre-tested and validated questionnaires that allow for comparisons with results from other studies. We used items from the Multiple Indicator Cluster Survey (MICS) water quality questionnaire to collect data on household water sources for drinking, cooking, hygiene and bathing, and on any pre-consumption treatment of drinking water [60]. Additionally, we used selected questions from the MICS water quality testing questionnaire to collect data regarding each water sample we collected [60]. The MICS is a program developed by the United Nations International Children’s Emergency Fund to regularly collected statistically sound and comparable household data and has been used in 120 countries globally [3, 60]. To assess households’ water insecurity, we applied the Household Water Insecurity Access Scale which is a module consisting of eight questions on the perception of water quantity and quality, feelings of worry or anxiety over water access and availability. The questions are answered using a 4-point Likert-type scale resulting in a total score ranging from 0 to 24, with higher scores indicating higher insecurity [61].

### Data analysis

We conducted descriptive analysis to explore and summarize the data, reporting proportions along with 95% confidence intervals (CI) using the Wilson Score Interval. While confidence intervals provide an inferential measure, no formal hypothesis testing or other inferential statistical analyses were applied. All data management and analysis were performed in RStudio, utilizing the binom package for CI estimation [62]. Figures were generated using RStudio, OmniGraffel, Adobe Illustrator and Microsoft Excel [62–65].

## Results

**Fig. 3:**
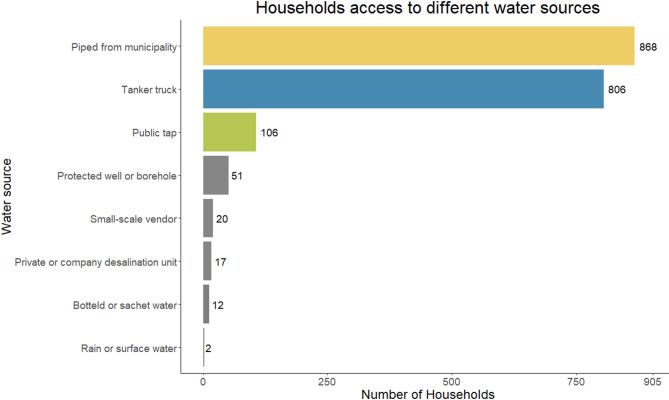
Households access to different water sources. We define access as any member from the household that was interviewed has mentioned the source for any of the water related questions on drinking, cooking and hygiene

We interviewed 2’291 people in 905 households and collected a water sample from 902 households. Two households refused to give a water sample and in one household no drinking water was present at the time of the interviews. Our survey shows that households in the Gaza Strip have access to different water sources, with almost all households having access to piped water from the municipality (95.9%, 95% CI: 94.4–97.0%) or tanker truck (89.1%, 95% CI: 86.9–90.9%) and using it for either drinking, cooking or hygiene purposes (Fig. 3). Only 3.0% (95% CI: 2.1–4.3%) of the households had access to a single water source while 86.9% (95% CI: 84.5–88.9%) had access to two water sources, 10.1% (95% CI: 8.3–12.2%) had access to three or four sources with one household having access to five sources.

**Fig. 4:**
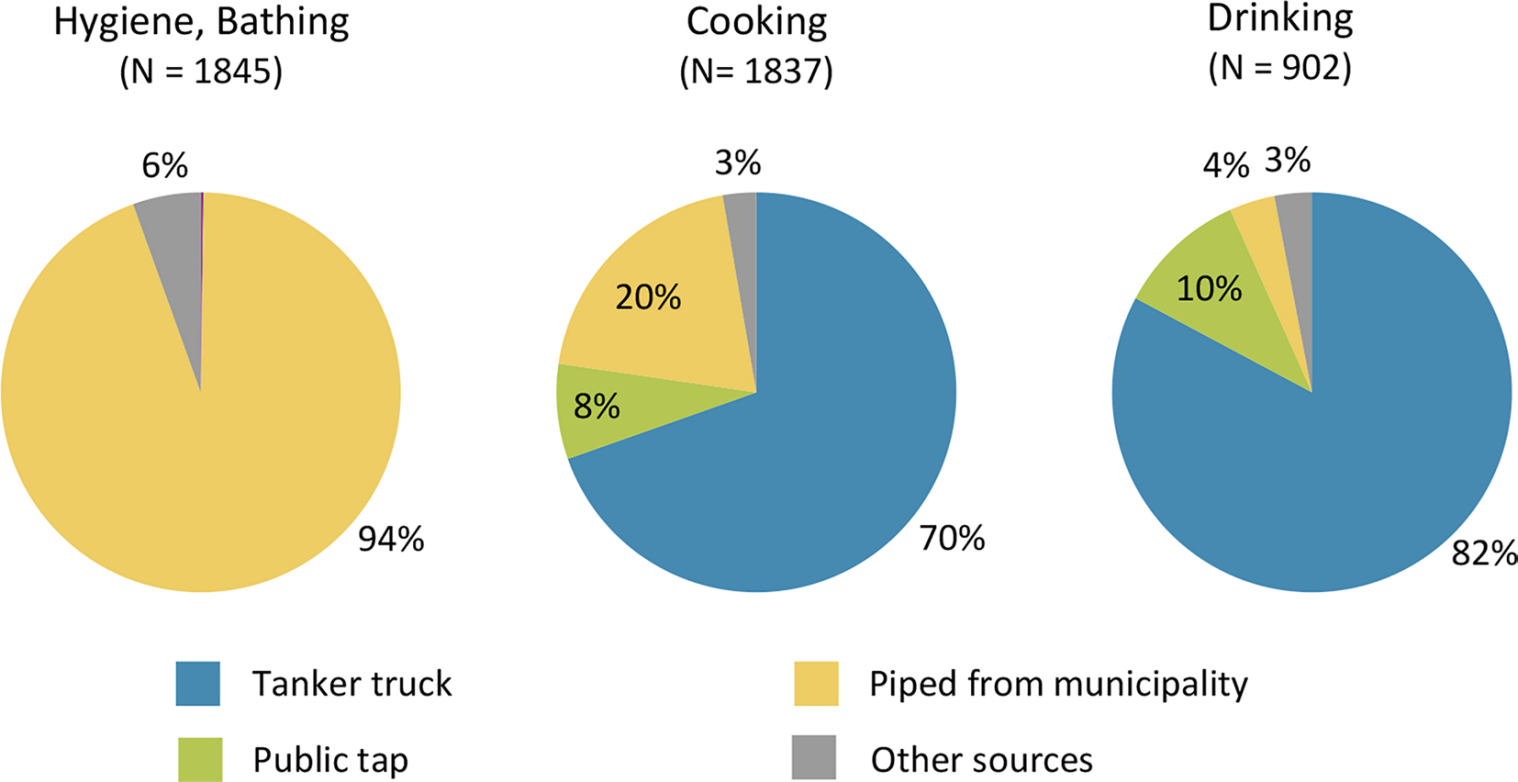
Water sources for different uses in the households. The water sources used for drinking show the sources of the water sample that was collected and only include one source per household. The sources for hygiene, bathing and cooking show the sources reported by the participants and can include more than one source per household

When we look at which water is used in the household for different purposes we can observe clear differences between the different tasks. For drinking water, most households use water from tanker trucks (82.3%, 95% CI: 79.6–84.6%), while water from public taps (10.4%, 95% CI: 8.6–12.6%) and piped water from the municipality (4.4%, 95% CI: 3.3-6.0%) are being less frequently used (Fig. 4). For cooking, tanker truck was still the most used water source (69.6%, 95% CI: 67.4–71.6%), followed by piped water from the municipality (20.0%, 95% CI: 18.3–21.9%) and public tap water (7.7%, 95% CI: 6.5-9.0%). For hygiene, bathing, showering and washing, 94.3% (95% CI: 93.1–95.2%) of the households reported using the piped water from the municipality (Fig. 4).

**Fig. 5:**
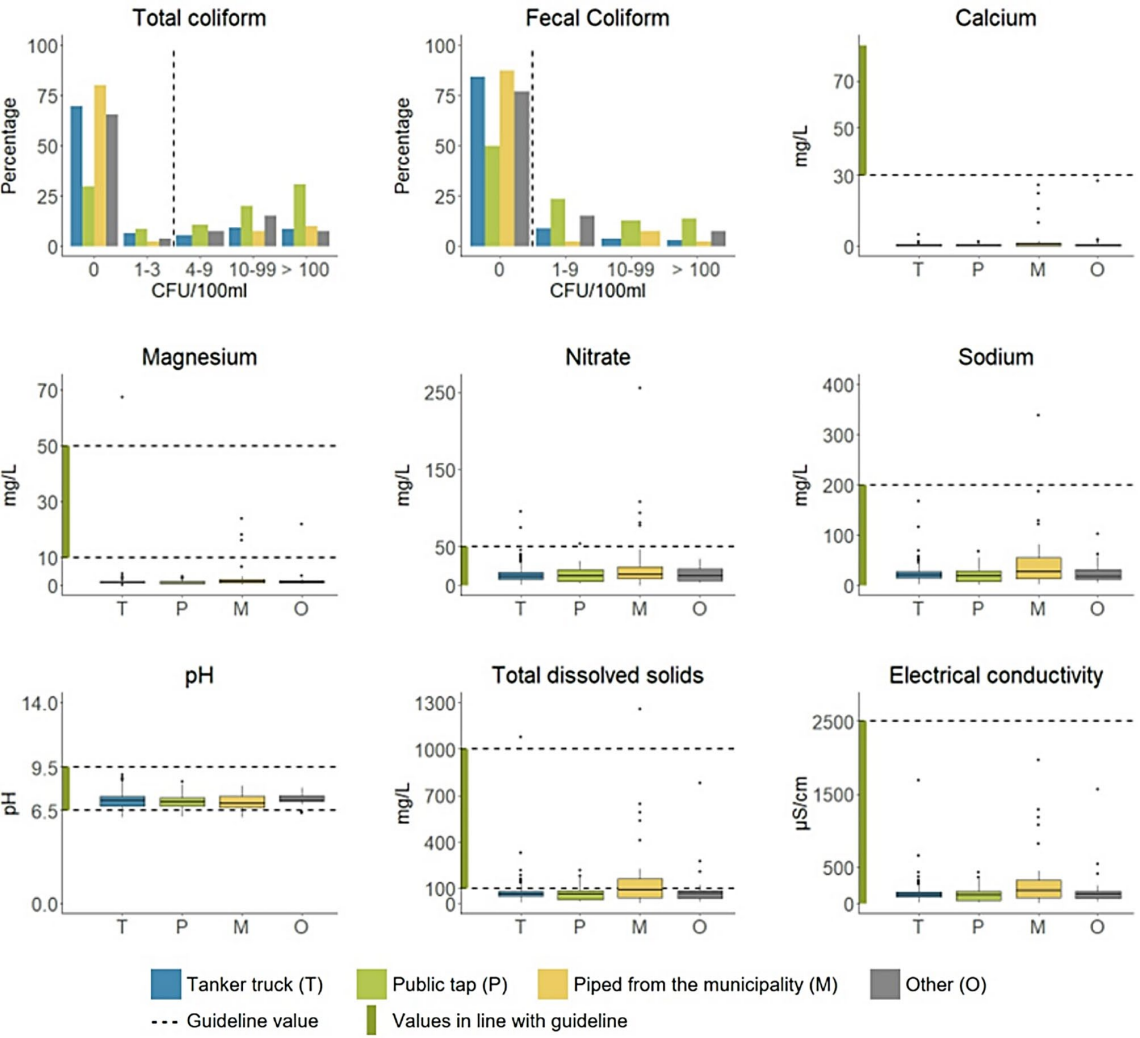
Distribution of water quality parameters by water source compared to Palestinian guideline values. Guideline values are described in Table 1 (*N* = 902). For some of the parameters outliers are not shown in the graph: calcium (*n* = 4), magnesium (*n* = 3), sodium (*n* = 2), total dissolved solids (*n* = 3) and electrical conductivity (*n* = 2)

Drinking water quality results are displayed in Fig. 5. Total coliforms were detected in 33.9% of the samples (95% CI: 30.9–37.1%), with 27.3% (95% CI: 24.5–30.3%) exceeding the Palestinian guideline of < 3 CFU / 100 ml. Fecal coliforms (Palestinian guideline: 0 CFU / 100 ml) were present in 19.3% of the samples (95% CI: 16.8–22.0%). Only tanker truck (15.6%, 95% CI: 13.2–18.4%) and municipality water (12.5%, 95% CI: 5.5–26.1%) had fecal coliform in less than 20% of the samples. In water samples from public taps, fecal coliform was detected in half of all samples. No fecal coliform was found in the samples from domestic desalination units (*n* = 4). Low mineral content (calcium < 30 mg/L and magnesium < 10 mg/L) was observed in 99.6% of the samples (95% CI: 98.9–99.8%), with only four samples exceeding both thresholds. Only two and five samples from the municipal water were in line with the guidelines for calcium and magnesium respectively, two samples from tanker trucks and one sample from water from a protected well (*n* = 5) had high enough levels of magnesium. All samples from all other sources had low calcium and mineral content. High nitrate (> 50 mg/L) and sodium (> 200 mg/L) levels were detected in 0.9% (95% CI: 0.5–1.7%) and 0.3% (95% CI: 0.1-1.0%) of the samples respectively. The pH level was outside of the guideline range (6.5–9.5) in 3.8% of the samples (95% CI: 2.7–5.2%), while 85.1% of the samples (95% CI: 82.7–87.3%) were outside the recommended range for total dissolved solids (100–1000 mg/L). The mean electrical conductivity was 154 µS/cm, ranging from 10.4 to 5750 µS/cm.

We conducted a water chain analysis with ten households, seven of which used tanker trucks and three used public taps as their main drinking water source. From each water chain, we collected between three and seven samples from different points along the water chain, resulting in a total of 55 samples. A schematic overview of the water chains and sampling locations is shown in Fig. 2. Fecal coliform was detected in 15 (27%, 95% CI: 17–40%) of the 55 samples. For two households with water from tanker trucks no coliform was detected in any of the samples along the water chain. In three water chains, contamination was detected already at the source (T1– T3 and P1– P3) and in three water chains samples contamination was found in the transport system (T4 and P4). Table 2 displays the number of samples collected at each location and the number of samples with microbial contamination.

**Table 2:** Water chain sample results. The sampling locations for the tanker truck (T1-T7) and public tap (P1-P6) water chain are described in more detail in Fig. 2

Sample		Total coliform		Fecal coliform	
Location	N	N Positive^a^	% (95% CI)^b^	N Positive^a^	% (95% CI)^b^
**All samples**	**55**	**23**	**42% (30–87%)**	**15**	**27% (17–40%)**
**Tanker truck**	**42**	**14**	**33% (21–48%)**	**10**	**24% (14–39%)**
T1	2	0	0% (0–66%)	0	0% (0–66%)
T2	4	0	0% (0–49%)	0	0% (0–49%)
T3	7	1	14% (3–51%)	0	0% (0–35%)
T4	11	2	18% (5–48%)	0	0% (0–26%)
T5	4	1	25% (5–70%)	1	25% (5–70%)
T6	6	4	67% (30–90%)	3	50% (19–81%)
T7	8	6	75% (41–93%)	6	75% (41–93%)
**Public tap**	**13**	**9**	**69% (42–87%)**	**5**	**39% (18–65%)**
P1	1	0	0% (0–79%)	0	0 (0–79%)
P2	3	2	67% (21–94%)	0	0 (0–56%)
P3	3	1	33% (6–79%)	0	0 (0–56%)
P4	2	2	100% (34–100%)	2	100% (34–100%)
P5	1	1	100% (21–100%)	1	100% (21–100%)
P6	3	3	100% (44–100%)	2	67% (21–94%)

^a^ Number of samples with total coliform (TC) or fecal coliform (FC) detected (> 0 CFU / 100 ml)

^b^ Percentage of TC resp. FC positive samples and the 95% confidence interval (CI)

In the survey, 90 of the 905 households reported that they usually treat the water before consumption. The treatment methods reported by those 90 households were the use of water filters (65.6%, 95% CI: 55.3–74.6%), boiling the water (27.8%, 95% CI: 19.6–37.8%), adding bleach or chlorine (4.4%, 95% CI: 1.7–10.9%) and letting the water stand and settle (3.3%, 95% CI: 1.1–9.3%). When asking specifically about the water that we sampled, 32 households had used filters, and 12 households had boiled the sampled water with only a total of 46 household applying any kind of treatment to the sampled water.

**Fig. 6:**
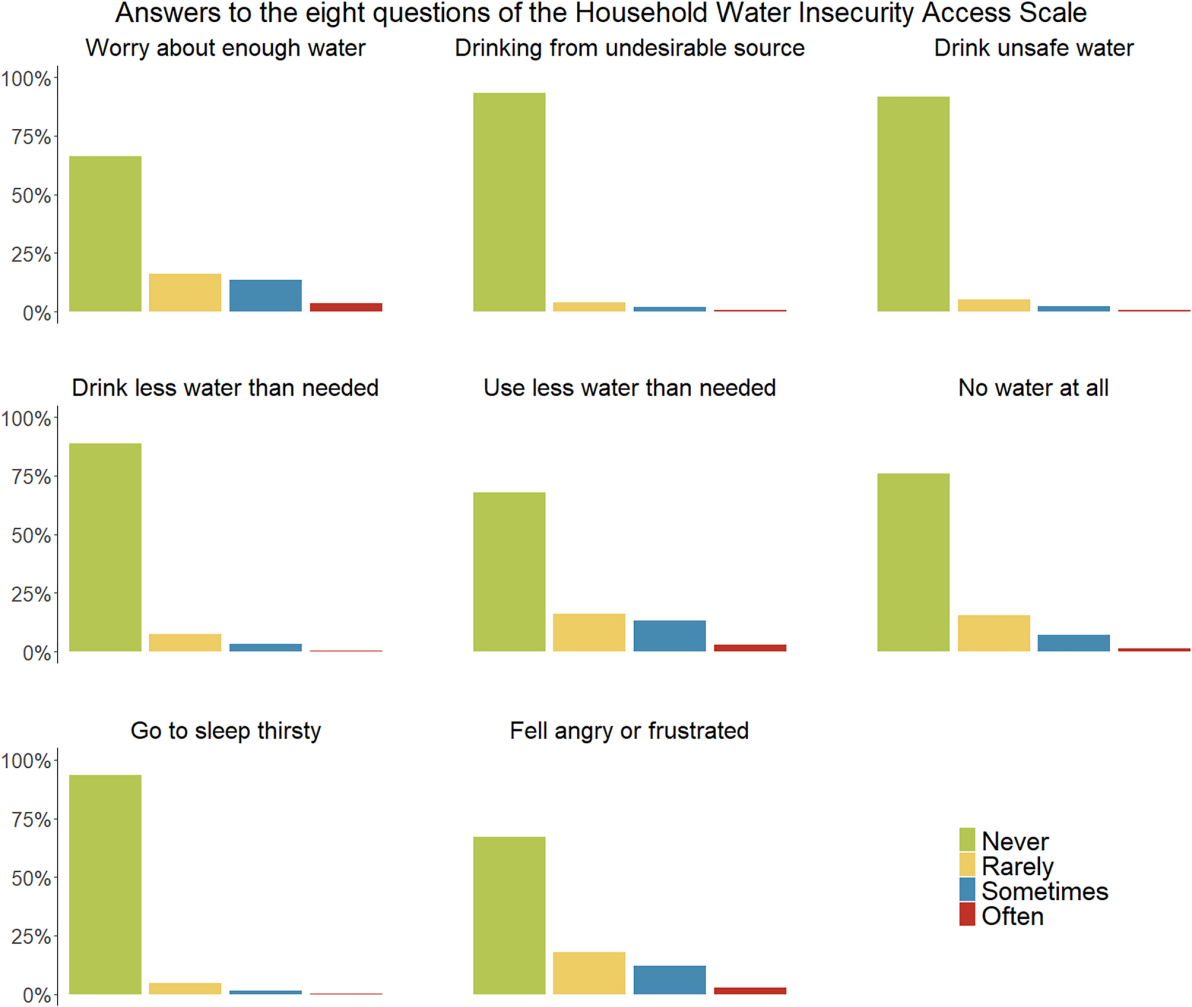
Participants answers to items of the Household Water Insecurity Access Scale (*N* = 2’291)

For all eight questions of the Household Water Insecurity Access Scale, fewer than 4% of participants reported experiencing that issue often. For three questions (worry about enough water, use less water than needed, and feel angry or frustrated about having enough water) between 14.9 and 17.4% answered with sometimes or often. Overall, 18.7% answered with sometimes or often to two or more of the questions and 74.7% answered all questions with never or rarely. More positively, over 90% reported never drinking from undesirable sources, drinking unsafe water, or going to sleep thirsty, and 50.8% answered all eight questions with never (Fig. 6).

A comparison of Household Water Insecurity Access Scale answers and scores between households using different drinking water sources is difficult due to the small number of households using a different source than tanker trucks. Households using water from the municipality had the lowest Household Water Insecurity Access Scale score, while households using a different source than the main three (tanker truck, public tap and municipality) had higher water insecurity than the other households.

## Discussion

We collected drinking water samples from 902 households in the Gaza Strip from January to March 2023. More households than expected primarily relied on tanker truck water from desalination plants for drinking. Consequently, we found low concentrations of minerals in almost all samples which bears the risk for a range of adverse health outcomes. The issue of microbial contamination was confirmed by our study, though it may be less of a concern than thought. The concerning findings in terms of water access and quality in the study area also resonated with the respondents of the survey, with approximately one-fifth of the participants reported some degree of water insecurity.

Our study found that almost all households use more than one water source, with piped water from the municipality and tanker truck water the most accessible sources. However, we observed a big difference in usage of the different sources. While almost all households reported using municipal water for hygiene and bathing, this changed drastically for cooking and especially drinking usage. This is in line with findings from other surveys in the Gaza Strip where the majority of households reported using tanker truck water [1, 3]. In 2020, approximately 70–80% of people were using water from desalination plants as their main source for drinking and cooking [3]. This has implications for quality control of drinking water. While the municipal water is managed and monitored by the municipality and authorities, there is less oversight and monitoring of private desalination plant and tanker trucks [5]. The question of why households choose to switch the water source is less clear. Previous studies reported that water source choices are driven more by aesthetic concerns, such as smell, taste and high salinity, than by microbiological water quality, even when the participants were aware of the difference in water quality [42–45]. While almost all water samples were within the recommended values for pH and electrical conductivity, previous studies reported an increase in salinity from 2002 to 2022 by 31% [33]. On the other hand, tanker truck water is generally more expensive than municipal water, adding a financial burden on households and potentially a barrier for household to change their water source. Reports from 2018 to 2022 show that tanker truck water was sold to consumers for approximately 7–9$ per 1,000 L, whereas the same quantity of municipal water cost less than 1$ [1, 5]. The findings suggest that a more resilient and centralized water system that can address aesthetic and quality concerns while remaining affordable for households should be prioritized post-war. Reducing household’s dependence on less-regulated private water sources should be central aim to ensure equitable access and sustainable water management.

We detected low minerals in almost all water samples, independent of water source. This is concerning, as numerous observational studies found that long-term exposure to low mineral content in drinking water is associated with adverse health outcomes [16, 22, 24, 66]. A recent study in the Gaza Strip found an association between a lack of magnesium in drinking water and osteoporosis [26] while several studies found an association between the consumption of desalinated water and cardio vascular health [17, 18, 20]. Yang and colleagues found potential associations between low calcium levels in drinking water and gastric, colorectal, pancreatic, colon and esophageal cancer [28–32]. This is important in the context of the Gaza Strip where a majority is consuming desalinated water. With continuous and increasing overuse of the aquifer, desalinated water will continue to play an important role as a drinking source in the future even after the current war. Because the water is demineralized in the desalination process, it is advised that important nutritional minerals such as calcium and magnesium are added before consumption [66]. However, the low levels of minerals in almost all our samples show that this is not the case in most desalination plants in the Gaza Strip which has been pointed out in previous reports [5]. When rebuilding the water system, remineralizing the water from desalination plants must be a priority by either strengthening the municipal water network where remineralization can be centralized on a system level or by increasing oversight over the desalination plants and tanker trucks.

Every fifth water sample in our study had fecal contamination and samples from various points in the water supply chain indicated that contamination happens at different points in the water chain. These findings are in line with multiple previous studies showing fecal contamination in households, but also in groundwater wells, desalination plants and tanker trucks in the Gaza Strip [4, 8, 67]. Our findings show that there is a serious risk for the transmission of water-borne diseases, from mild infections like acute diarrhea to severe conditions such as chronic diarrhea, typhoid, and potentially cholera [10]. This is even more of a concern when looking at the presence of antimicrobial resistant bacteria in seawater, humans, hospital surfaces and tap water as well as in chicken farms [13–15, 68] as well as in drinking water collected in our study which have been published elsewhere [69]. The treatment of infections is complicated by the wide spread presence of antimicrobial resistance in the Gaza Strip [12]. While interventions at the production and distribution level (e.g. desalination plants and tanker trucks or wells and pipes) are important to reduce the risk of contamination, the potential risks at the household level also have to be addressed. Storage at the household level (e.g. roof top tanks or containers in the kitchen) pose a risk to contamination [2] and piped water from the municipality is considered a safer distribution option than tanker trucks [3, 70]. While tackling fecal contamination in drinking water would also reduce potential antimicrobial resistance bacteria in drinking water it is essential to acknowledge that antimicrobial resistance is a multi-sectoral issue that has to be addressed simultaneously in different sectors and by different actors in a joint effort to make an impact. This is also important in light of the reconstruction of the water system where a silo thinking should be avoided in order to best tackle system wide challenges such as antimicrobial resistance.

Contrary to most previous studies, we detected high levels of nitrate and sodium only in a few samples. While previous studies and monitoring reports from the authorities sampling at groundwater wells detected high nitrate levels [2, 6, 33, 54, 71], other studies sampling at desalination plants found nitrate levels comparable to our results [71]. The drinking water samples in our study originated mainly from desalination plants and the groundwater was mixed with other sources before distribution. This could explain the low levels of nitrate levels detected and might also lower the public health concerns about high nitrate levels in the drinking water. Effects from the war, such as chemicals and heavy metal, together with contamination from untreated wastewater and continued seawater intrusion could render the aquifer unsafe for drinking water and increase reliance on desalination plants [72–75]. Nonetheless, nitrate and sodium remain potentially harmful chemicals that have to be monitored regularly as long as groundwater is used in agriculture and as drinking water.

Our study demonstrates that water insecurity is a problem in many households in the Gaza Strip underlining this issue which was already identified in previous reports [1]. Insecurities exist around water availability and cost while the water has been reported to taste salty and thus water quality is perceived as poor and not good for drinking [1, 76, 77]. This underlines the importance of monitoring and controlling not only potentially harmful parameters such as bacteria and chemicals, but also parameters such as pH, salinity and total dissolved solids, influencing the taste and appearance of water [42–45]. A recent report with adolescents showed that water insecurity increased drastically since the beginning of the war with nearly 90% of the participants now being highly water insecure [78]. This underlines the importance of water infrastructure during reconstruction efforts. At the same time, it is essential that the rebuilding process promotes health and addresses the shortcomings of the pre-war water system, avoiding a replication of past issues.

### Strengths and limitations

One of the strengths of our study is the water sampling at the household level. We sampled the same water that the members of the household drank, without any storage, transportation or treatment steps in between the sample site and the consumption. This allows us to test the water quality of the water consumed and to better understand potential health risks.

The main limitation of our study is the cross-sectional design of the water sampling. As water sources can be changed over time and water quality fluctuates, our measurement only gives us an indication of water quality issues at the time of the sample collection. While we did try to take water samples from the main drinking water of each household, it is also possible that some households recently changed their source or were currently using an alternative source. Finally, while we asked about the sources used for different tasks in the household (i.e. drinking, cooking, hygiene and bathing), we did not ask for the reason behind these choices, limiting our ability to interpret these findings.

## Conclusions

Our study highlights various challenges within the drinking water sector in the Gaza Strip which should be considered during the reconstruction of the water system. Given the pre-war overuse of the aquifer leading to high salinity, the extensive destruction of water infrastructure, and the very likely contamination of groundwater due to the ongoing war, the long-term viability of the aquifer as a drinking water source is increasingly uncertain. In this context, desalination plants will play an even greater role in meeting water demand. However, while desalination addresses water scarcity, its widespread use also introduces new challenges, particularly the low mineral content of desalinated water and high costs of tanker truck water. The low mineral content of the water can have long-term health implications if not properly managed and consequently, it will be important to prioritize remineralization along with safe transportation and storage of water to mitigate microbial contamination. However, the effectiveness of measures to improve water quality largely depends on the population’s utilization of these water sources. As shown in our study, most households had access to multiple sources and choose their usage based on their specific needs. To enable informed decisions regarding water sources, the public must have access to real-time monitoring of municipal water quality. Such transparency would enable individuals to choose safer water sources, reducing economic burdens and insecurities related to water accessibility and quality. Moving forward, reconstruction efforts should not only restore infrastructure but also integrate sustainable solutions that ensure both the safety and health benefits of desalinated water, while addressing the broader structural issues that have challenged water access in the Gaza Strip.

## Electronic supplementary material

Below is the link to the electronic supplementary material.

Supplementary Material 1

## Data Availability

The data that support the findings of this study are available on request from the corresponding author.

## Abbreviations

CI: Confidence interval
HIA: Health impact assessment
ICRC: International Committee of the Red Cross
MICS: Multiple Indicator Cluster Survey
WHO: World Health Organization
