# Supplementary material for: Drinking water access and quality in the Gaza Strip prior to 7 October 2023 and implications for reconstruction
Source: Environ Health. 2025 Jul 1;24:41. doi: 10.1186/s12940-025-01191-6 (PMC12220596; doi:10.1186/s12940-025-01191-6)
Supplement: Supplementary file 1 — Supplementary Material 1 [file 12940_2025_1191_MOESM1_ESM.docx]

# Additional File 1 to Brugger et a. 2025 - Drinking water access and quality in the Gaza Strip prior to 7 October 2023 and implications for reconstruction.

Supplementary Table ST 1: Analytical methods of the Coastal Municipalities Water Utility laboratory.
The Coastal Municipalities Water Utility laboratory used the analytical methods for the required parameters according to the American Public Health Association, American Water Works Association and Water Environment Federation’s Standard methods for the examination of water and wastewater (21st edition) (57).

| **Parameter** | **Abbreviation** | **Unit** | **Analytical method** | **Methods No.** | **Detection limit** | **Device Name** |
| --- | --- | --- | --- | --- | --- | --- |
| Total coliform | TC | CFU/100mL | Membrane technique, with M-Indo agar media and incubation at 35^o^C for 24 hours. | SM: 9221-B | more than 0.00 |  |
| Fecal coliform | FC | CFU/100mL | Membrane technique, with M-FC agar media and incubation at 45^o^C for 24 hours. | SM: 9221-E | more than 0.00 |  |
| Calcium | Ca^+2^ | mg/L | Argentometric, EDTA titration | SM: 3500 Ca-B | more than 0.00 |  |
| Magnesium | Mg^+2^ | mg/L | Calculation from and Ca^+2^ Hardness | SM: 3500 Mg-B | more than 0.00 |  |
| Nitrate | NO_3_-N | mg/L | Ultraviolet Spectrophotometric Method | SM: 4500 NO3-B | more than 0.00 | WTW photoLab 6000 |
| Sodium | Na^+^ | mg/L | Flame emission photometric | SM: 3500 Na-B | up to 60 | Sherwood M410  Flame Photometer |
| pH | pH | pH units | Electrometric Method, pH meter. | SM: 4500-D | 0 to 14 | Hach sensIon MM150 |
| Hardness |  | mg/L | Argentometric, EDTA titration | SM: 2340-C | more than 0.00 |  |
| Electrical conductivity | EC | µS/cm | Electrometric (EC meter) | SM: 2510-B | more than 0.00 | Hach sensIon MM150 |
